# 812. Bacterial endosymbionts are frequently found among Mucorales clinical isolates. Should we screen for their presence in patients with Mucormycosis?

**DOI:** 10.1093/ofid/ofad500.857

**Published:** 2023-11-27

**Authors:** Dora Edith Corzo Leon, Jamie Harrison, Jessie Uehling, Luis Esaú López-Jácome, Andy Borman, Elizabeth Ballou

**Affiliations:** MRC Centre for Medical Mycology at University of Exeter, EXETER, England, United Kingdom; MRC Centre for Medical Mycology at University of Exeter, EXETER, England, United Kingdom; Oregon State University, Corvallis, Oregon; Instituto Nacional de Rehabilitación "Luis Guillermo Ibarra Ibarra", Mexico City, Distrito Federal, Mexico; UK National Mycology Reference Laboratory, Bristol, England, United Kingdom; MRC Centre for Medical Mycology at University of Exeter, EXETER, England, United Kingdom

## Abstract

**Background:**

Soil-associated Mucorales fungi frequently host bacterial endosymbionts that produce damaging endotoxins important for plant pathology. Our work and clinical reports suggest an important and unanticipated role for endosymbionts in human disease. The endosymbiont *Mycetohabitants rhizoxinica* was associated with bacteriemia in an immunocompromised individual where its host *Rhizopus microsporus* caused mucormycosis. Our data demonstrate the endosymbiont *Ralstonia pickettii* can impact virulence of its *R. microsporus* host. Together, these data suggest Mucorales endosymbionts can influence both fungal and bacterial infections; however, their frequency and diversity in Mucormycosis is unknown. This project investigates the frequency and diversity of bacterial endosymbionts in Mucorales clinical isolates.

**Methods:**

Mucorales isolates from mucormycosis patients via the UK National Mycology Reference Laboratory and the Instituto Nacional de Rehabilitacion, Mexico, were identified by ITS1/2 PCR or MALDI-TOF. Fungal isolates (YPG agar, 5 days, 30 ^o^C) were subjected to phenol/chloroform DNA extraction. Bacterial presence was assessed by 16s RNA PCR, and resulting bands identified by nanopore sequencing. In parallel, WGS of total DNA confirmed bacterial presence and identity. Bacteria were also physically isolated from homogenized fungal mycelia (nutrient broth, 80rpm, 37^o^C, 4 days) harvested under sterile conditions. Bacteria from supernatants (LB, Blood agar, 30 and 37 ^o^C ± 5% CO_2_, 4-10 days) were identified by 16s RNA PCR and Nanopore sequencing.

**Results:**

In total, 27 Mucorales isolates (*R. arrhizus/delemar* n=7; *R. microsporus* n=6; *Lichtheimia corymbifera* n=4; *L. ramosa* n=3; *Mucor circinelloides* n=4; others n=3) were evaluated for bacteria presence. 17/27 (62%) were 16s RNA positive. Culture identified 6 bacterial species (*R. pickettii, Niallia circulans, Peribacillus simplex, Roseomonas mucosa, Micrococcus luteus, Staphylococcus pasteuri*). Mucorales isolates originated from cutaneous 11/27 (41%); respiratory 8/27 (29%); and sinus-orbital diseases 7/27 (26%).

**Conclusion:**

The presence of bacterial endosymbionts in clinical isolates of Mucorales is frequent, diverse and can potentially influence fungi in causing disease among individuals at risk.

**Disclosures:**

**All Authors**: No reported disclosures

